# AI-assisted system improves the work efficiency of cytologists via excluding cytology-negative slides and accelerating the slide interpretation

**DOI:** 10.3389/fonc.2023.1290112

**Published:** 2023-11-23

**Authors:** Hui Du, Wenkui Dai, Qian Zhou, Changzhong Li, Shuai Cheng Li, Chun Wang, Jinlong Tang, Xiangchen Wu, Ruifang Wu

**Affiliations:** ^1^ Department of Obstetrics and Gynecology, Peking University Shenzhen Hospital, Shenzhen, China; ^2^ Institute of Obstetrics and Gynecology, Shenzhen Peking University-The Hong Kong University of Science and Technology (PKU-HKUST) Medical Center, Shenzhen, China; ^3^ Shenzhen Key Laboratory on Technology for Early Diagnosis of Major Gynecologic Diseases, Shenzhen, China; ^4^ Department of Computer Science, City University of Hong Kong, Hong Kong, Hong Kong SAR, China; ^5^ Suzhou Ruiqian Technology Company Ltd., Suzhou, China

**Keywords:** HPV, cervical cancer screening, artificial intelligence, slide interpretation, low-resource areas

## Abstract

Given the shortage of cytologists, women in low-resource regions had inequitable access to cervical cytology which plays an pivotal role in cervical cancer screening. Emerging studies indicated the potential of AI-assisted system in promoting the implementation of cytology in resource-limited settings. However, there is a deficiency in evaluating the aid of AI in the improvement of cytologists’ work efficiency. This study aimed to evaluate the feasibility of AI in excluding cytology-negative slides and improve the efficiency of slide interpretation. Well-annotated slides were included to develop the classification model that was applied to classify slides in the validation group. Nearly 70% of validation slides were reported as negative by the AI system, and none of these slides were diagnosed as high-grade lesions by expert cytologists. With the aid of AI system, the average of interpretation time for each slide decreased from 3 minutes to 30 seconds. These findings suggested the potential of AI-assisted system in accelerating slide interpretation in the large-scale cervical cancer screening.

## Introduction

Cervical cancer is threatening women’s health and caused 342,000 deaths worldwide in 2020 (1). Screening plays an important role in eliminating cervical cancer, such as diagnosing precancerous cervical intraepithelial neoplasia (CIN) that can be surgically eliminated to prevent the incidence of cervical cancer (2, 3). However, there are disparities of cervical cancer screening globally (4, 5). Self-sampling has dramatically ameliorated inequity of human papillomavirus (HPV) testing especially in resource-limited settings (6–9). In contrast, cervical cytology remains an issue in low-resource regions due to the shortage of cytologists (10, 11).

Emerging studies indicated the potential of artificial intelligence (AI) system for cervical cytology (12–16). For instance, Cheng et al. applied a recurrent neural network-based whole slide image (WSI) classification model to achieve high specificity and sensitivity for slide classification (14). Nevertheless, most of prior reports assessed the potential of AI system in classifying cervical lesions (12–15). Besides to the diagnosis of cervical lesions, it is important to lessen the dependence of cervical cytology on professionals in resource-limited settings. Given the indispensable role of cytologists in cervical cancer screening, it is imperative to reduce the number of cytologist-interpreted slides and to shorten the interpretation time for each slide.

Our study aimed to evaluate how AI-assisted system improved the work efficiency of cytologist-based cytology. To fulfill this goal, well-annotated cervical slides were applied to develop the model of slide classification. Then we assessed the feasibility of AI system in excluding NILM(Negative for Intraepithelial Lesion or Malignancy) slides. Subsequently, the efficiency of slide interpretation was evaluated for cytologists with and without the aid of AI system. These findings should have the potential to promote the implementation of cervical cytology in China.

## Materials and methods

### Automated staining and microscopic imaging of cervical exfoliated cells

We applied a liquid-based sedimentation cytology approach RQLCT1000 (Ruiqian co. ltd, Jiangsu) to achieve stained slides. Then, the automated slide scanner RQ1000 (Ruiqian co. ltd, Jiangsu) using continuous array scanning technology was applied to rapidly generate multi-depth images. The scanning process included two stages: 10X and 20X microscope scanning and multi-depth scanning as well as seamless layer fusion via Z-stack technology (17).

### Image annotation

Three experimental clinicians and two expert pathologists from tertiary hospitals annotated scanned slides, adhering to TBS-2014 guidelines (18). In detail, digital images were divided into three non-repetitive sets for annotation by distinct medical professionals. Annotation cells, which results were agreed by two experts, were integrated into a standardized database for robust data management.

### Cell detection and classification

A 10× image acquisition system was used for Pap test AI detection to identify single cells and cell clusters. Cell clusters were characterized by closely packed cells without easily distinguishable cytoplasm (**Supplementary Figure 1**). Meanwhile, each cell cluster should include more than three cells.

We then classified cells via two modules: cell primary screening and cell classification module. The cell primary screening module applied Yolov5 as the basic framework, and the cell classification module utilized ResNet as the basic framework (19–21). Among them, the cell primary screening module was used for detecting all suspicious lesioned cells in the image to ensure a high recall rate of detected diseased cells. The cell classification module further screened and classified diseased cells on the basis of the cell primary screening module to improve the accuracy of detecting diseased cells.

The dataset of the cell primary screening module was composed of annotated cells, that were classified into NILM(Negative for Intraepithelial Lesion or Malignancy), HSIL(High-Grade Squamous Intraepithelial Lesion), LSIL (Low-Grade Squamous Intraepithelial Lesion), and ASU(Atypical Squamous Cells of Undetermined Significance) types.

The dataset of the cell classification module was composed of the detected positive cell images of the cell primary screening module, wherein the ‘false’ diseased cells detected by the cell primary screening module were regarded as NILM, and the detected ‘true’ diseased cells were regarded as lesion-positive.

### Slide classification

To determine if the slide was lesioned, we first ranked all single cells and cell clusters based on lesion probability. Afterwards, we calculated the average probability of top 10 single cells as well as the average probability of top 5 cell clusters. Then scatter plotting was conducted for all slides to determine the cutoff of probability, using the average of top 10 single cells as x-axis and the average of top 5 cell clusters as y-axis. And SVM classifier was applied to determine the degree of lesion, such as ASU and HSIL (22).

### Statistic analysis

To assess the feasibility of AI system in excluding NILM slides, we calculated the percentage of AI-reported NILM slides in the validation group. Then we compared it to cytologist-interpreted results.

For slides in the validation group, the interpretation time of cytologists was recorded for two types of slide interpretation: with and without the aid of AI system. To compare the differences of consumed time in slide interpretation, the average of the interpretation time was calculated by the formula: the total of consumed time/slide number. In addition, cytologist-reported results were also compared between two interpretation models.

## Results

### Automatic AI system to perform cell staining and imaging

**Figure 1:**
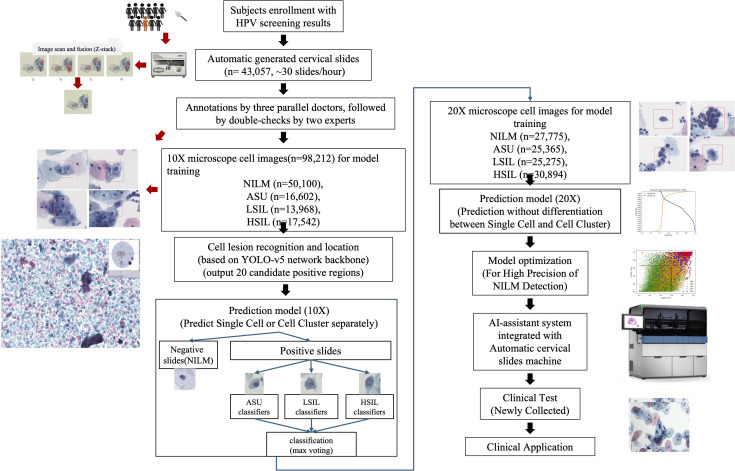
The workflow of automatic AI-assisted cervical cytology.

We devised an integrated approach for the automated diagnosis of cervical lesions, utilizing the cytology of cervical exfoliated cells (**Figure 1**). All experiments were seamlessly integrated into an automated and cohesive process, encompassing the staining of exfoliated cells and multi-depth scanning fusion imaging.

Within this automatic system, we totally scanned 43,057 cervical slides in two hospitals within 74 days (10 running hours/day), resulting in an average production rate of nearly 30 high-quality images per hour. Then we selected 5,000 high-quality slides for developing cell classification model and the remaining 31,753 high-quality slides were applied to assess the performance of developed models.

### Performance of AI-system in classifying cervical cells

Based on 5,000 high-confidence slides, 98,212 high-quality 10× images of individual cells were chosen, being annotated as NILM (n=50,100), ASU (n=16,602), LSIL (n=13,968), and HSIL (n=17,542) (**Table 1**). Additionally, we selected 64,087 10× high-quality images for cell clusters, which were annotated as NILM (n=34,775), ASU (n=9,062), LSIL (n=9,552), and HSIL (n=10,698) (**Table 1**). The confusion matrix showed that the accuracy of NILM prediction from single cell and cell cluster was 81.40% and 88.85%, respectively. The highest accuracy were found for HSIL prediction (90.0% and 85.0% for single cell and cell cluster), compare to the ASU (42.54% and 37.17% for single cell and cell cluster) and LSIL(68.23% and 65.75% for single cell and cell cluster) (**Table 2**).

**Table 1:** The types and distribution of 10× single cell and cell cluster.

Types		Total	Train set	Validation set	Test set
**Single Cell**	**NILM**	50,100	35,070	10,020	5,010
	**ASU**	16,602	11,622	3,320	1,660
	**LSIL**	13,968	9,778	2,793	1,397
	**HSIL**	17,542	12,280	3,508	1,754
**Cell Cluster**	**NILM**	34,775	24,343	6,955	3,477
	**ASU**	9,062	6,344	1,812	906
	**LSIL**	9,552	6,687	1,910	955
	**HSIL**	10,698	7,489	2,139	1,070

**Table 2:** The confusion matrix of 10× single cell and cell cluster.

Prediction Labels		True Labels			
		NILM	ASU	LSIL	HSIL
Single Cell	NILM	81.40%	31.98%	6.74%	4.68%
	ASU	8.0%	42.54%	15.05%	3.93%
	LSIL	2.25%	11.42%	68.23%	1.39%
	HSIL	8.34%	14.06%	9.98%	90.0%
Cell Cluster	NILM	88.85%	23.32%	6.13%	8.96%
	ASU	1.58%	37.17%	20.82%	2.68%
	LSIL	1.98%	32.72%	65.75%	3.37%
	HSIL	7.59%	6.78%	7.31%	85.0%

The same methods were applied in 20× microscope classification, but combination of cell clusters and single cells were used to train the classification model. As the accuracy of NILM remained at 81.49%, there was a reduction in misclassification ratio from ASU, LSIL and HSIL to NILM by 10.61%, 2.71% and 4.45% (**Table 3**).

**Table 3:** The distribution and confusion matrix of 20× cervical cell image.

Types	Total	Train set	Validation set	Test set	True Labels			
					NILM	ASU	LSIL	HSIL
NILM	27,775	19,443	5,555	2,777	81.49%	10.61%	2.71%	4.45%
ASU	25,365	17,756	5,073	2,536	9.79%	63.04%	18.24%	3.96%
LSIL	25,275	17,693	5,055	2,527	6.33%	18.81%	72.88%	2.31%
HSIL	30,894	21,626	6,179	3,089	2.39%	7.54%	6.17%	89.27%

**Figure 2:**
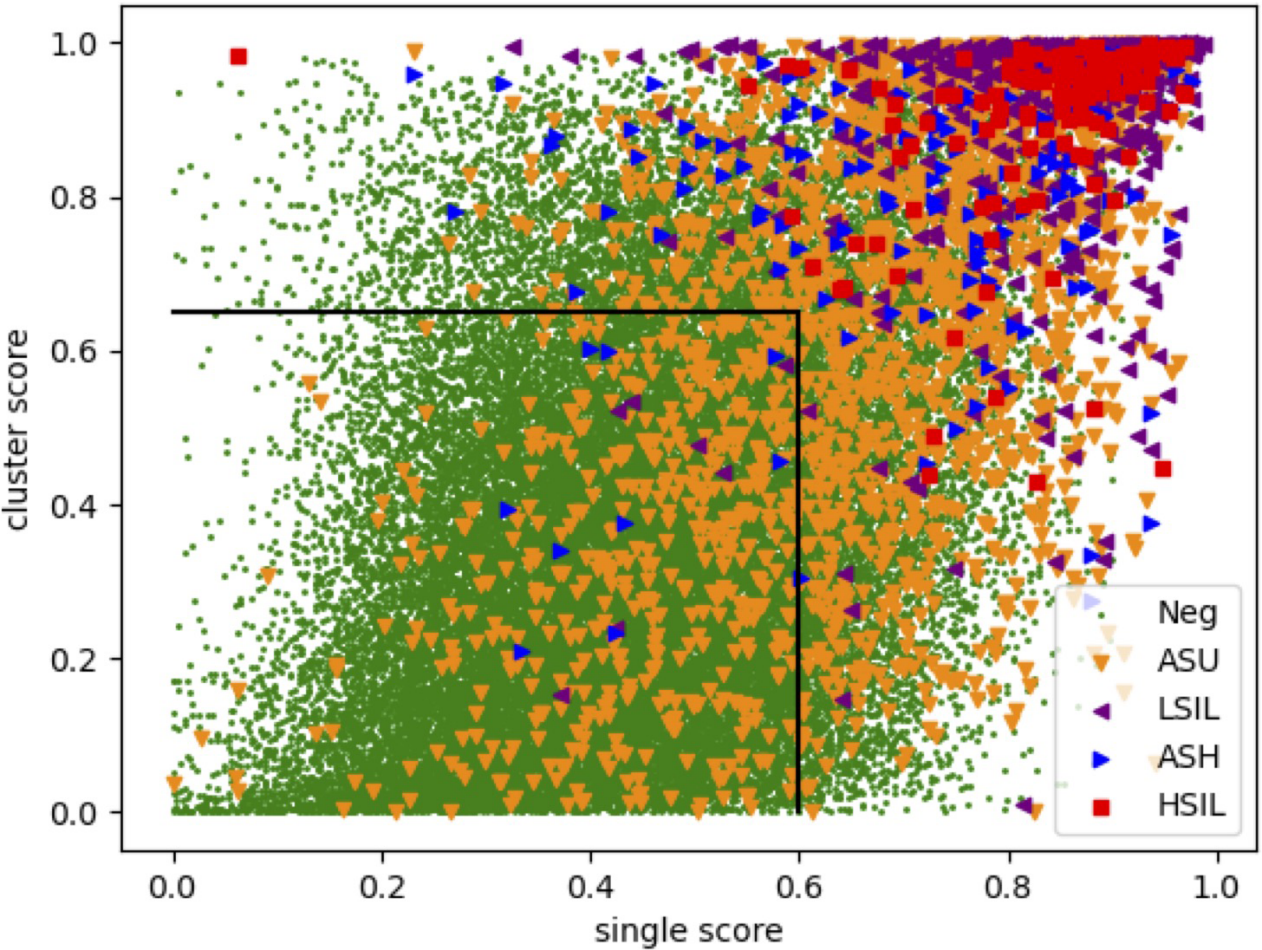
Scatter plot for slide assessment based on single cell and cell cluster scores. Each specific represents a slide and the colors represent expert-conducted diagnosis of the slide. X-axis and y-axis represent the average of probability for top 10 single cells and top 5 cell clusters, respectively.

To achieve the higher accuracy of NILM prediction, we calculated both the average probability of the top 10 single cells and the average probability of the top 5 cell clusters. By applying a cutoff of 0.6 for single cell scores and 0.65 for cell cluster scores (**Figure 2**), we achieved the highest accuracy in NILM prediction.

### Performance of AI system in excluding cytology-negative slides and improving the efficiency of the slide interpretation

We further applied our AI-system on 31,753 slides in the validation groups. AI system reported 29,625 slides with NILM among which 98.27% (29,113/29,625) were also diagnosed as NILM by two expert cytologists who reviewed slides together and provided a consensus result. For remaining 512 AI-reported NILM slides, 1.67% (496/29,625) and 0.05% (16/29,625) were diagnosed as ASU and LSIL by cytologists. None of AI-reported NILM slides was diagnosed as HSIL.

Besides to accurately exclude cytology-negative slides, our AI system shortened the interpretation time for cytologists. Based on AI-assisted system, cytologists only need to interpret the top 20 cells which were ranked by the lesion probability. Therefore, the average interpretation time for each slide can decrease from 3 minutes to 30 seconds. And we observed no differences of interpretation results for 979 AI-reported lesion-positive slides between whole slide image and AI-provided top 20 cells, including 129 HSIL, 137 ASH and 713 LSIL slides.

## Discussion

Cervical cancer screening play an important role in eliminating cervical cancer (2, 3). Despite the potential of AI-assisted system in facilitating cervical cytology, professionals play essential roles in near future. Nevertheless, there is a deficiency in assessing the improved work efficiency of cytologists and the lessened dependence on professionals based on AI-assisted system. Our study evaluated the exclusion of cytology-negative slides and shortened interpretation time for cytologists with the aid of AI system.

Our large-scale cervical cancer screening projects indicated that nearly 80% of cervical slides were cytology-negative (23–26). Therefore, it can dramatically decrease the workload of cytologists if the AI-system can exclude these cytology-negative slides with high accuracy. In this study, we identified the potential of AI-assisted system in excluding cytology-negative slides, thus decreasing the number of slides to be interpreted by cytologists. Nearly 70% of analyzed slides were reported as NILM by AI-assisted system. Among 29,625 AI-reported NILM slides, 98.27% were diagnosed as NILM. The remaining 1.72% were diagnosed as ASU as well as LSIL by cytologists. Thus, the risk of missing HSIL slides is low when applying the AI system to exclude cytology-negative slides.

Besides to excluding about 70% cytology-negative slides, AI system can shorten the interpretation time of AI-reported positive slides (27). In prior, it took about 3 minutes for to interpret a slide. With the aid of the AI-assisted system, cytologists only need to interpret the top 20 cells which were ranked by the lesion probability. Therefore, the average interpretation time for each slide can decrease from 3 minutes to 30 seconds.

The limitations of our study included the retrospective design. And it was not tested in real resource-limited settings. In addition, it was completed with two steps: preparing slides then conducting slide scanning and AI-assisted cytology. Thus, it would bring additional burden and decrease work efficiency in resource-limited settings. However, our team and other researchers are testing one-stop machine, which can automatically perform high-throughput slide preparation, staining, imaging and AI-assisted cytology. Then it can further improve the feasibility and efficacy of cervical cytology in large-scale cervical cancer screening projects. And this should ensure the equity of cervical cancer screening and precancer/cancer treatment for underserved communities in China.

In summary, AI-assisted system can improve the work efficiency of cytologists, such as decreasing the number of slides to be interpreted and shortened the time of slide interpretation. Additionally, automatic sample processing and AI-assisted cytology can lessen the dependence of cytology on medical resources. And this should increase the coverage of cervical cancer screening in low-resource regions.

## Data availability statement

The original contributions presented in the study are included in the article/**Supplementary Material**. Further inquiries can be directed to the corresponding authors.

## Ethics statement

This study was approved by the Ethics Committee of Peking University Shenzhen Hospital (registration number: 2023-116).

## Author contributions

HD: Conceptualization, Funding acquisition, Resources, Writing – original draft. WD: Methodology, Visualization, Writing – original draft. QZ: Methodology, Writing – original draft. CL: Methodology, Software, Writing – original draft. SL: Methodology, Writing – original draft. CW: Data curation, Resources, Validation, Writing – original draft. JT: Data curation, Validation, Writing – original draft. XW: Conceptualization, Supervision, Writing – review & editing. RW: Conceptualization, Resources, Supervision, Writing – review & editing.
