# Supplementary figures and images for: AI-assisted system improves the work efficiency of cytologists via excluding cytology-negative slides and accelerating the slide interpretation
Source: Front Oncol. 2023 Nov 23;13:1290112. doi: 10.3389/fonc.2023.1290112 (PMC10701732; doi:10.3389/fonc.2023.1290112)

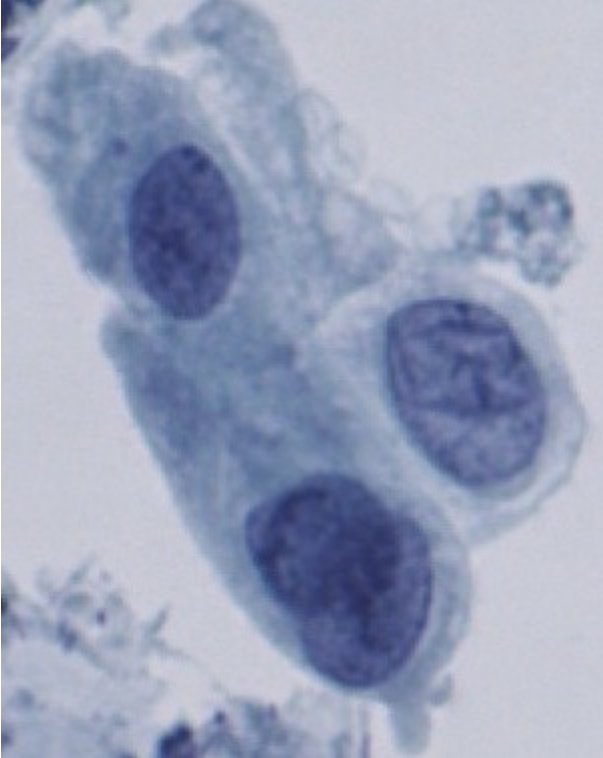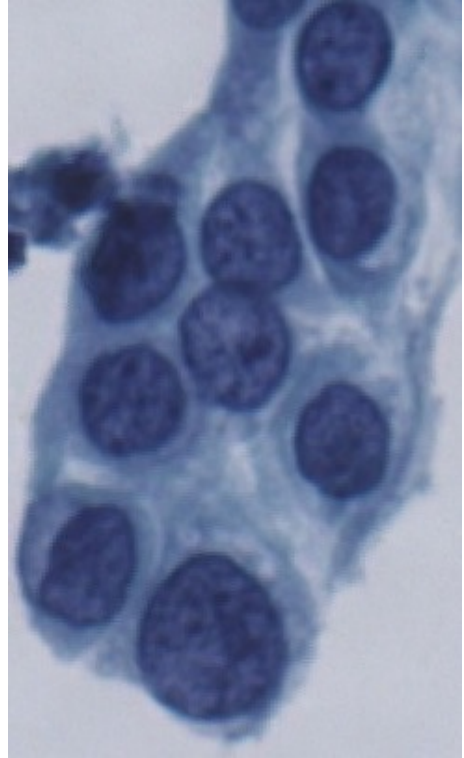

Supplement: Supplementary file 1 [file Image_1.pdf]
